# Global mapping of institutional and hospital-based (Level II–IV) arthroplasty registries: a scoping review

**DOI:** 10.1007/s00590-023-03691-y

**Published:** 2023-09-28

**Authors:** Aikaterini Zgouridou, Eustathios Kenanidis, Michael Potoupnis, Eleftherios Tsiridis

**Affiliations:** 1 Academic Orthopaedic Department, Aristotle University Medical School, General Hospital Papageorgiou, Ring Road Efkarpia, 56403 Thessaloniki, Greece; 2 Centre of Orthopaedic and Regenerative Medicine (CORE), Center for Interdisciplinary Research and Innovation (CIRI)-Aristotle University of Thessaloniki (AUTH), Balkan Center, Buildings A & B, 10th km Thessaloniki-Thermi Rd, P.O. Box 8318, 57001 Thessaloniki, Greece

**Keywords:** Arthroplasty replacement, Joint registry, Arthroplasty registry, Hospital-based registry, Regional registry, Registry level

## Abstract

**Purpose:**

Four joint arthroplasty registries (JARs) levels exist based on the recorded data type. Level I JARs are national registries that record primary data. Hospital or institutional JARs (Level II–IV) document further data (patient-reported outcomes, demographic, radiographic). A worldwide list of Level II–IV JARs must be created to effectively assess and categorize these data.

**Methods:**

Our study is a systematic scoping review that followed the PRISMA guidelines and included 648 studies. Based on their publications, the study aimed to map the existing Level II–IV JARs worldwide. The secondary aim was to record their lifetime, publications’ number and frequency and recognise differences with national JARs.

**Results:**

One hundred five Level II–IV JARs were identified. Forty-eight hospital-based, 45 institutional, and 12 regional JARs. Fifty JARs were found in America, 39 in Europe, nine in Asia, six in Oceania and one in Africa. They have published 485 cohorts, 91 case-series, 49 case–control, nine cross-sectional studies, eight registry protocols and six randomized trials. Most cohort studies were retrospective. Twenty-three per cent of papers studied patient-reported outcomes, 21.45% surgical complications, 13.73% postoperative clinical and 5.25% radiographic outcomes, and 11.88% were survival analyses. Forty-four JARs have published only one paper. Level I JARs primarily publish implant revision risk annual reports, while Level IV JARs collect comprehensive data to conduct retrospective cohort studies.

**Conclusions:**

This is the first study mapping all Level II–IV JARs worldwide. Most JARs are found in Europe and America, reporting on retrospective cohorts, but only a few report on studies systematically.

## Introduction

Joint arthroplasty registries (JARs) are databases that report the outcomes of joint arthroplasties (JAs). Their primary goal is the JAs quality improvement [1], and they are considered the gold standard source for informed medical decision-making. There are four levels of JARs based on the type of data recorded. Level I JARs record basic data, Level II demographic and comorbidity data, Level III patient-reported outcome data and Level IV imaging and radiographic data. Most well-known national JARs collect type I data because further data collection is expensive. Fewer regional, institutional, or hospital-based JARs collect more detailed patients’ data (type II–IV) [1, 2].

National arthroplasty registries (Level I) report primary data on patients and procedures using revision arthroplasty as the endpoint. They collect large data volume nationally, reporting annual survival outcomes and revision risk of specific implants [3, 4]. However, national JARs reports are extensive and interpreted with difficulty by clinicians having little statistical training. The reports’ interpretation may also be misleading due to the absence of more comprehensive registry data (type II–IV) [5, 6].

On the other hand, Level II–IV regional or hospital-based registries collect smaller volumes but more inclusive data correlating efficiently radiologic or patient history data with arthroplasty outcomes. These JARs may complement national JARs, allowing further scrutiny and deeper causative correlation of JAs failure, improving outcomes [5]. Currently, an attempt to record the hip and knee JARs in Europe is being made [6]. However, a worldwide list of hospital-based JARs does not exist, and their contribution to assessing arthroplasty results remains unclear.

Level I JARs have been thoroughly researched, but there is a lack of information on the importance of Level II–IV JARs in the literature. To accurately evaluate and classify the more specific and patient-centric data they provide, we require a comprehensive inventory of both institutional and hospital-based JARs. Knowing the quantity and location of these JARs and the number and variety of publications they produce can enhance our comprehension of their value and necessity.

The present study is a systematically performed scoping review. The primary aim of this study was to map the existing institutional and hospital-based (Level II–IV) JARs worldwide and their lifetime. The secondary aim was to record their lifetime, publications’ number and frequency and recognize differences with national JARs. Countries with national and hospital-based JARs were also recorded.

## Materials and methods

Our study is a systematic scoping review that followed the PRISMA 2020 statement [7].

### Search strategy

A systematic review of published articles from several databases such as MEDLINE (PubMed), Cochrane Database of Systematic Reviews and Clinical Trials by the U.S. National Library of Medicine was conducted from conception to July 2022. The following keywords and Mesh terms were utilized with “AND” or “OR”: “arthroplasty, replacement, knee,” “arthroplasty, replacement, hip,” “arthroplasty, replacement, ankle,” “arthroplasty, replacement, shoulder,” “registries,” “arthroplasty registry,” “joint registry,” “regional registry,” “hospital registry”,” registry level,” “national registry.” The authors created the keywords, drawing on their own experience, and employed different names to refer to the term "registry". They did not involve any input from a librarian.

### Inclusion and exclusion criteria

Specific inclusion criteria were the following: i) randomized (RCTs) and non-randomized control trials, prospective and retrospective cohorts, case series and comparative studies, (ii) studies involving adult patients (> 18 years) that underwent elective total joint arthroplasty (TJA), (iii) studies evaluating joint arthroplasty outcomes based on Level II–IV JARs data (regional, institutional or hospital-based arthroplasty registries), (iv) studies providing extractable data (studies that have organized and fully structured data that can be extracted from the manuscript).

On the other hand, studies were excluded if they i) reported national type I JARs data, (ii) used non-arthroplasty registries data (hospital discharge or other ailments registries), (iii) were narrative reviews, letters to the editor, editorial comments, meta-analysis or systematic reviews related to the topic, (iv) were conducted in animals or cadavers, (v) were written in a non-English language, (vi) had no full-text available.

### Data extraction

The searched papers with abstract information were managed in Mendeley to remove duplicated citations. The remaining studies were screened independently by two authors. Firstly, titles and abstracts were screened using the search strategy to fulfil the inclusion criteria. The data extraction process was done by the two authors independently. The final extracted data were cross-checked. A third senior author resolved any disagreement.

### Data synthesis

Data synthesis was performed and analyzed by the same two authors that recorded the following information for JARs: i) the location (country, city, hospital name); (ii) the quality of reported studies (study type, methodology, population and other characteristics) (iii) their lifetime calculated from the time of the first and last found publication and (iv) if the countries of hospital-based registries had also a national registry. Differences in the published information between national and hospital-based registries were also evaluated.

## Results

### Search results

**Fig. 1:**
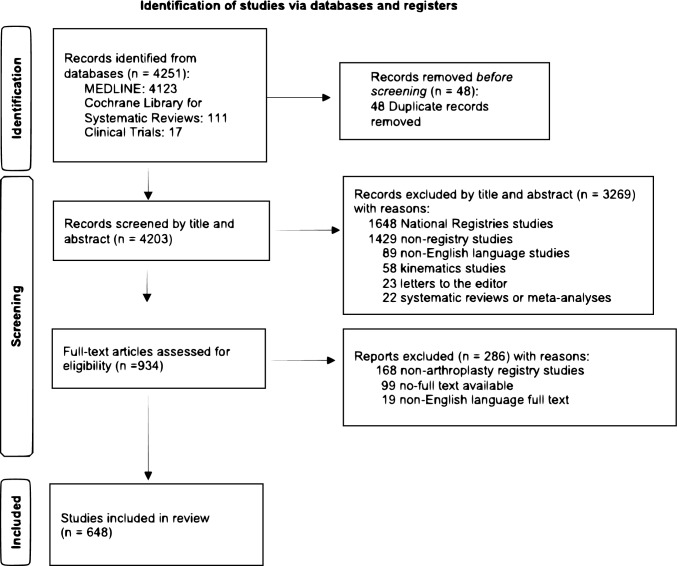
PRISMA 2020 flow diagram of search strategy

The initial electronic search yielded 4251 studies. After eliminating 48 duplicated studies, 4203 were reviewed on their title and abstract. According to our inclusion and exclusion criteria, 3269 records were excluded based on title and abstract, and 934 papers were deemed suitable and screened in the full article text. Finally, 648 studies were included in this systematic review. The flow diagram of the search strategy is shown in Fig. 1.

### Demographics and patient characteristics, study type, design & primary aim

The included studies were published from 1997 to 2022 [8–12]. The sample size of the studied population ranged from 9 to 84,998 patients [13, 14]. Almost 60 per cent of the patients were women. The follow-up of patients varied from three months to twenty-five years [15, 16].

According to the study type, 485 (74.85%) were cohorts [2, 3, 8–12, 14–316], 91 (14.04%) were case-series [13, 317–406], 49 (7.56%) were case–control studies [407–455], nine (1.39%) cross-sectional studies [456–464], eight (1.23%) protocols for registry-based studies [465–472] and six (0.93%) randomised control trials (RCT) [473–478]. Three hundred twenty-seven cohort studies were retrospective [2, 8, 11, 14, 17, 18, 21–26, 29–34, 36, 38, 41, 42, 44–46, 54–56, 64–71, 83–91, 106–118, 123, 137–146, 155–190, 200–217, 233–266, 273, 281–308, 311–315, 479–590], and 158 were prospective [9, 10, 12, 15, 16, 19, 20, 27, 28, 35, 37, 39, 40, 43, 47–53, 58–63, 73–82, 92–105, 119–136, 147–154, 191–199, 210, 218–232, 267–272, 274–280, 309, 310, 316, 591–629]. In thirty-seven cohort studies, a comparison between two cohorts was made [57, 72, 147, 188–191, 200, 233, 265, 309–316, 479–482, 526–528, 560–567, 630–633].

Almost half of the included papers studied the patient-reported outcome measures (PROMs), the Quality-of-Life Years (QALYs) and the intra- and postoperative complications rate. In detail, the primary outcome in 149 (23%) studies were PROMs and QALYs [18, 19, 23, 34, 40, 55, 59, 61, 68, 81, 92, 99, 100, 108, 111, 117, 124, 127, 129, 135, 137, 139, 150, 153, 161, 168, 170, 173, 182, 187, 191, 193, 196–200, 206, 207, 210, 212, 215, 217, 222, 223, 225, 227, 236, 242, 244, 245, 263, 265, 267, 268, 273, 275–278, 281, 283, 300, 301, 307, 310, 314, 339, 352, 353, 357, 368, 378, 382, 395, 396, 400, 401, 403, 405, 408, 412, 417, 425, 426, 434, 439, 440, 445, 446, 449, 460–463, 481, 483–485, 487, 492, 494, 499, 501, 512, 513, 516, 523, 525, 528, 530, 533, 542, 546, 548, 549, 564, 565, 574–576, 579, 587, 589–591, 593–595, 601, 602, 605–609, 612–614, 619, 621, 622, 625, 628, 629, 634–636], while in 139 (21.45%) studies were surgical complications (i.e., infections, fractures, thrombosis etc.) [9, 13, 15–17, 20, 21, 25, 45, 58, 63, 66, 69, 73, 77–80, 84, 85, 88, 101–105, 107, 109, 110, 113, 119, 121, 125, 130, 133, 146, 147, 152, 157, 159, 160, 162, 163, 166, 172, 174, 175, 180, 183, 194, 195, 202, 204, 209, 213, 234, 237, 239, 240, 250, 252, 254, 257, 264, 266, 269–271, 279, 282, 287, 289, 290, 295, 298, 306, 311, 312, 316, 317, 319, 327, 334, 336, 341, 354, 356, 365, 366, 370, 377, 381, 388, 392, 397, 398, 409, 413, 416, 422, 427, 432, 437, 441, 442, 444, 451, 452, 474, 475, 478, 480, 488, 498, 500, 504, 509, 520, 522, 529, 534, 536, 545, 547, 550, 554–556, 558, 560, 561, 568, 596, 597, 600, 630, 637–639]. Eighty-nine studies (13.73%) evaluated postoperative patients’ clinical outcomes [11, 27, 47, 50, 62, 74, 83, 91, 95–97, 106, 112, 114, 120, 128, 134, 149, 151, 176, 178, 181, 185, 188, 190, 192, 203, 205, 208, 210, 216, 221, 224, 229, 230, 235, 238, 247, 261, 286, 291, 292, 296, 302, 303, 305, 348, 349, 369, 372, 373, 375, 379, 380, 386, 387, 391, 411, 415, 419, 420, 424, 429, 430, 436, 454, 493, 496, 497, 503, 521, 526, 531, 537, 553, 559, 566, 567, 570, 573, 584, 616, 617, 633, 640–643], 34 (5.25%) postoperative radiographic outcomes [35, 56, 116, 148, 167, 169, 171, 246, 248, 274, 284, 304, 309, 324, 335, 343, 363, 364, 376, 447, 450, 453, 459, 464, 477, 489, 490, 514, 517, 535, 539, 572, 618, 644] and 46 (7.10%) studies assessed the efficacy of a specific implant [22, 24, 41–44, 71, 72, 86, 87, 118, 126, 165, 184, 201, 219, 233, 241, 243, 256, 262, 297, 325, 332, 337, 347, 359, 394, 410, 423, 431, 443, 479, 506, 541, 544, 580, 586, 598, 615, 624, 627, 632, 645, 646]. Seventy-seven (11.88%) studies were survival analyses [2, 10, 26, 37, 38, 46, 64, 67, 70, 115, 122, 136, 154, 155, 164, 177, 179, 189, 211, 231, 232, 251, 260, 272, 280, 288, 293, 308, 321, 329, 331, 340, 342, 344–346, 351, 360, 361, 371, 385, 402, 414, 421, 433, 438, 482, 491, 495, 502, 507, 508, 511, 515, 518, 519, 524, 527, 532, 540, 551, 557, 563, 569, 581–583, 585, 588, 592, 599, 603, 611, 631, 647, 648] but 21 (3.24%) studied the long-term arthroplasty outcomes [29, 48, 49, 82, 93, 141, 218, 228, 285, 322, 383, 384, 407, 458, 505, 543, 562, 623, 626, 649]. Besides, 17 (2.62%) studies compared different surgical techniques [28, 53, 144, 145, 186, 253, 338, 358, 362, 374, 390, 428, 538, 552, 578, 650, 651] and seven (1.08%) studies evaluated various levels of surgeons’ experience [30, 33, 36, 214, 255, 294, 299], while in 18 (2.78%) studies, a prediction of pre- or postoperative risk factors was made [14, 39, 52, 54, 98, 131, 132, 143, 355, 367, 389, 404, 435, 448, 456, 457, 604, 620]. Finally, 15 (2.31%) were cost analysis studies [57, 65, 75, 90, 138, 140, 142, 156, 158, 226, 315, 326, 328, 399, 486], 13 papers (2%) studied the patients’ mortality rate [31, 60, 76, 258, 259, 313, 320, 323, 333, 418, 577, 652, 653], 12 (1.85%) studies offered general registry information [8, 51, 89, 123, 220, 249, 318, 330, 350, 393, 571, 610], nine (1.39%) were protocols [465–473] and two (0.31%) genetic studies [32, 476].

### Global mapping of Level ΙΙ–IV registries

**Fig. 2:**
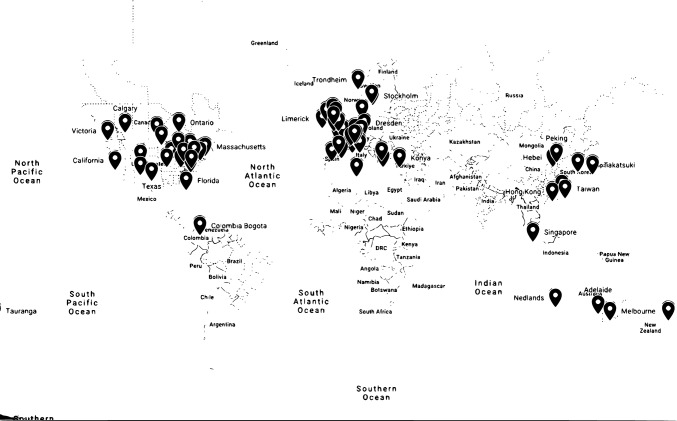
Global distribution of institutional and hospital-based (Level II–IV) arthroplasty registries. The figure was created in Google Maps (online). Each color point represents an arthroplasty registry (Level II–IV) in this area

105 Level II–IV registries were identified. Forty-eight (45.71%) were hospital-based, forty-five (42.86%) were institutional, and twelve (11.43%) were regional JARs. Tables 1, 2, and 3 show the distribution of the included JARs per continent. Specifically, 50 (47.62%) Level II–IV JARs were found in America (USA:44, Canada:5, SouthAmerica:1), 39 (37.14%) in Europe (Switzerland:7, UK:7, France:5, Germany:4, Italy:3, Spain:2, Greece:2, Ireland:2, Sweden:2, Norway:1, Denmark:1, Austria:1, Scotland:1, Turkey:1), nine (8.57%) in Asia (China:4, Taiwan:1, Japan:1, Hong Kong:1, Korea:1, Singapore:1), six (5.71%) in Oceania (Australia:5, New Zealand:1) and one (0.95%) in Africa (Tunisia) (Tables 1, 2 and 3). Some countries have more than one institutional JARs in different cities, while others have only one hospital-based arthroplasty JAR. The global geographic distribution of the included type II–IV JARs is depicted in Fig. 2.

**Table 1:** Mapping of hospital-based arthroplasty registries in Oceania, Asia and Africa continent

Continent	Country	Region	JAR
Oceania	Australia	Adelaide	(Revision Knee) Repatriation General HS Adelaide [590]
		Melbourne	1. St. Vincent’s HS Melbourne (SMART) [65, 158, 197, 214, 228, 303, 347, 397, 460, 465, 470, 472, 478, 525, 536, 558, 571, 607]
			2. Alfred HS [341]
		Nedlands	Hollywood HS H&K [34]
		Victoria	(Barwon) St John of God HS [399]
	New Zealand	Tauranga	Regional Tauranga Public HS [386]
Asia	China	Hebei	Hebei Medical University [204]
		Fujian	First Affiliated HS of Fujian Medical University [555]
		Peking	1. Knee Peking Union Medical College HS [370]
			2. Chinese People’s Liberation Army [539]
	Hong Kong	Hong Kong	Institutional Queen Mary HS [282, 367, 471, 554, 617, 622]
	Japan	Takatsuki	(Hip) Takatsuki General HS [359]
	Korea	Korea	Korean Hip (KHR) [489]
	Republic of China	Taiwan	Chang Gung Memorial HS [83, 422, 475, 557]
	Singapore	Singapore	Singapore General HS [108, 124, 168, 192, 227, 229, 245, 253, 273, 277, 284, 434, 436, 440, 443, 466, 467, 513, 538, 546, 579, 591, 595, 602, 608, 612, 616, 621, 623, 640, 641, 649, 657]
Africa	Tunisia	Tunisia	Local TKA Kassab Orthopaedic Institute [388]

*JAR* Joint Arthroplasty Registry, *H&K* Hip and Knee, *HS* Hospital, *S&E* Shoulder and Elbow, *THR* Total Hip Registry, *TJA* Total Joint Arthroplasty, *TKA* Total Knee Arthroplasty

[] Numbers in parentheses are the relevant references

**Table 2:** Mapping of hospital-based arthroplasty registries in Europe

Country	Region	JAR
Austria	Innsbruck	Tirol Landeskrankheitstalten GmbH [396, 423]
Denmark	Hvidovre	Copenhagen University HS [278, 587]
France	Livet	Livet HS [334]
	Lyon	1. Hopital Prive Jean Mermoz Shoulder [398]
		2. Hôpital Edouard Herriot [280, 361]
	Nice	Hopital Pasteur 2 Shoulder, Universite Cote d’Azur [398]
	Paris	SoFCOT Group [41, 102, 104, 115, 130, 340, 657]
Germany	Dresden	1. ORTHOTEP University HS Carl Gustav Carus [109, 619]
		2. Dresden Hip Surgery Registry [52, 196]
	Heidelberg	University of Heidelberg [410]
	Regensburg	Regensburg University [198, 255, 292, 296, 299]
Greece	Athens	General HS KAT [155, 384, 646]
	Thessaloniki	Arthroplasty RG Thessaloniki (ART) [2, 12, 406, 455, 647]
Italy	Emilia-Romanga	Register of Orthopaedic Prosthetic Implant (RIPO) [24, 26, 39, 43, 57, 145, 164, 189, 231, 288, 297, 468, 476, 482, 491, 502, 506, 508, 527, 540, 562, 563, 569, 585, 592, 597, 599, 624, 630, 645]
	Milano	Italian Arthroplasty RG (RIAP) [87, 378, 395, 451, 570]
	Toscana	Santo Stefano HS [442]
Ireland	Limerick	University HS Limerick [431]
	Northern Ireland	Musgrove Park HS [32]
Norway	Trondheim	H&K, St Olavs HS [176, 628]
Scotland	Edinburgh	University Edinburgh, Scotland [528]
Spain	Catalonia	Catalan Arthroplasty Register (RACat) [203, 313, 556, 350, 469, 658]
	Madrid	University HS Gregorio Maranon [226]
Sweden	Stockholm	Department of Orthopedics Södersjukhuset [105, 175]
	Uppsala	Uppsala University HS [262]
Switzerland	Bern	Shoulder, Orthopädie Sonnenhof [444]
	Geneva	Geneva Arthroplasty RG (HUG) [51, 103, 131, 219, 267, 271, 310, 311, 458, 594, 620, 625, 626]
	Lausanne	Lausanne Uni HS—CHUV [584, 638, 394]
	Liesta	Kantonsspital Baselland Liesta [239, 342]
	St. Gallen	TKA RG, Kantonsspital St. Gallen [351, 428, 439, 514, 528, 575, 614]
	Zurich	1. Balgrist Uni HS Zürich [91, 453]
		2. Schulthess Shoulder Arthroplasty RG (SAR) [248, 382, 387, 391, 496, 545, 561, 610]
Turkey	Konya	Selcuk University Medical Faculty [368]
United Kingdom	Bistrol	Avon Knee RG [29, 36]
	Derby	Royal Derby Hospital [402]
	Leicester	Trent [9, 20–22, 27, 53, 218, 256, 276, 329]
	London	Royal National Orthopaedic HS RG [177]
	Newcastle	Freeman Joint RG [111, 178, 349, 364, 613, 642]
	SouthWest London	SouthWest London Elective Orthopaedic Centre [117, 139, 324, 337, 461–463]
	Wringtington	North West Wrightington HS [30, 33]

*JAR* Joint Arthroplasty Registry, *H&K* Hip and Knee, *HS* Hospital, *RG* Registry, *S&E* Shoulder and Elbow, *TJA* Total Joint Arthroplasty, *TKA* Total Knee Arthroplasty

[] Numbers in parentheses are the relevant references

**Table 3:** Mapping of hospital-based arthroplasty registries in America

Country	State-region	JAR
Canada	Calgary	ABJHI [534]
	Ontario	1. OJRR [55, 328, 330] 2. Southwestern Ontario [23]
	Toronto	1. St Michael’s HS [611, 433] 2. Toronto Western HS [15, 40, 48–50, 54, 68, 107, 603, 411, 456]
	Winnpeg	University of Manitoba [300, 366, 477, 487, 492]
South America	Colombia Bogota	HS Universitario Fundación Santa Fe de Bogotá [220, 291, 376, 615]
United States of America	California	1. CJRR [146, 150, 215, 317, 353, 365] 2. Saint Vincent Medical Center [38] 3. University California, San Francisco [18, 392] 4. Stanford University Medical Center, San Francisco, Redwood City [438]
	Colorado	1. Colorando JRR [446] 2. Steadman Philippon Research Institute Registry, Vail [457]
	Connecticut	1. CJRI [17] 2. University Connecticut Health Center, Farmington [275, 314, 316]
	Florida	1. FOI [419] 2. Center for Advanced Orthopedics Larkin, Miami [200, 355, 426] 3. Joint RG Mercy HS, Miami [408] 4. Knee Registry St. Vincent’s Healthcare, Jacksonville [347]
	Illinois	1. American Hip Institute, Westmont [435, 549, 553, 445, 605] 2. Rush Uni (Shoulder), Chicago [209, 302, 535, 565, 588]
	Kentucky	Kentucky University, Lexington [144, 212, 216, 304, 352, 358, 441, 531]
	Massachusetts	1. New England Baptist, Tufts [296–306] 2. PAR [307–310] 3. Harris, Massachusetts General HS [311–316, 591–599] 4. FORCE-TJR Massachusetts University [594, 595]
	Michigan	1. MARCQI [14, 186, 202, 205, 221, 254, 369, 373, 381, 404, 437, 520, 637, 651] 2. Retrieved Orthopedic, Beaumont Health [336]
	Minnesota	1. HealthEast [28, 37, 42, 70, 72, 331, 414] 2. Minneapolis Veteran’s Affairs (VA) [185] 3. Minnesota University [136, 160, 345]
	Missouri	Barnes-Jewish HS Washington University [56, 133, 141, 162, 184, 213, 230, 285, 400, 421, 448, 644, 657]
	New Mexico	(JRR) New Mexico Orthopaedics [16]
	New York	1. CHKR [74] 2. NY-Presbyterian HS Columbia University [75] 3. Sinai HS, Baltimore [86] 4. HS for Special Surgery [11, 19, 59, 61, 63, 98, 113, 116, 119, 121, 128, 148, 156, 167, 170, 173, 181, 187, 193, 206–208, 210, 217, 222, 223, 233, 235, 236, 238, 241–243, 246, 261, 265, 266, 268, 270, 279, 281, 283, 298, 301, 307, 479, 483–485, 488, 493, 494, 499, 501, 512, 517, 521, 530, 533, 541, 542, 483–485, 573, 580, 589, 632, 635, 643, 339, 346, 357, 372, 377, 389, 405, 412, 420, 425, 427, 429, 430, 432, 447, 473, 601, 618, 658–665] 5. Mount Sinai [147, 504] 6. Mayo Clinic, Rochester [8, 13, 25, 31, 45, 58, 60, 62, 64, 67, 69, 73, 76–82, 84, 85, 88, 92–95, 106, 110, 118, 120, 122, 125–127, 129, 132, 134, 135, 137, 138, 140, 142, 143, 152, 154, 157, 159, 163, 166, 169, 174, 179, 180, 190, 194, 195, 201, 232, 234, 240, 244, 247, 249, 251, 252, 257–260, 263, 264, 269, 289, 290, 293, 305, 306, 308, 312, 320–323, 325–327, 500, 507, 509–511, 515, 518, 519, 524, 529, 532, 543, 548, 550, 551, 559, 568, 576, 578, 582, 583, 586, 652, 653, 631, 650, 509–511, 321–323, 325–327, 332, 333, 335, 338, 348, 354, 356, 363, 371, 374, 375, 380, 383, 385, 390, 407, 409, 416, 449, 450, 666–671]
	North Carolina	1. OrthoCarolina H&K, Charlotte [46, 165, 172, 237, 272] 2. OrthoCarolina S&E Center, Charlotte [537] 3. Ankle, Duke University Medical Center, Durham [672]
	Ohio	1. Joint Implant Surgeons Practice RG [171, 250, 295,505, 344, 360, 379, 498, 503, 418, 673] 2. EVEREST [47] 3. Cleveland Clinic Foundation [188, 319, 362, 474] 4. University of Cincinnati Medical Center, Cincinnati [572]
	Pennsylvania	1. TKA Dartmouth-Hitchcock Medical Center, Lebanon [294] 2. Thomas Jefferson University HS, Rothman Institute of Orthopaedics, Philadelphia [35, 44, 66, 325, 581, 627]
	Texas	1. FORI (Shoulder) [153, 182, 183, 424, 526, 566, 567, 604] 2. Texas Southwestern University [211, 459]
	Virginia	OrthoVirginia [287, 547]

*ABJHI* Alberta Bone and Joint Health Institute, *CHKR* Center for Hip and Knee Replacement Joint Registry, *CJRI* Connecticut Joint Replacement Institute, *CJRR* California Joint Replacement Registry, *FOI* Florida Orthopaedic Institute, *Shoulder, FORI* Fondren Orthopedic Research Institute, *H&K* Hip and Knee, *HS* Hospital, *JAR* Joint Arthroplasty Registry, *JRR* Joint Replacement Registry, *MARCQI* Michigan Collaborative Quality Initiative, *NY* New York, *RG* Registry, *OJRR* Ontario Joint Replacement Registry, *PAR* Partners Massachusetts Registry, *S&E* Shoulder and Elbow, *TJA* Total Joint Arthroplasty, *TKA* Total Knee Arthroplasty

[] Numbers in parentheses are the relevant references

Table 4 shows the number of publications and the time of the first and last publication for those JARs with more than one published study. Forty-four JARs have published only one paper, and 74 JARs have a publication lifetime of fewer than five years. The “Mayo Clinic Total Joint Registry” has been reporting studies for twenty-five consecutive years, followed by the “Trent” JAR for twenty-one years and the “Register of Orthopaedic Prosthetic Implant (RIPO) of Emilia-Romagna region” for twenty years. The “Mayo Clinic Total Joint Registry” has published 149 papers from 1997 to 2022 [8, 650], including 120 cohort studies [8, 25, 31, 45, 58, 60, 62, 64, 67, 69, 73, 76–82, 84, 85, 88, 92–97, 99–101, 106, 110, 112, 114, 118, 120, 122, 123, 125–127, 129, 132, 134, 135, 137, 138, 140, 142, 143, 152, 154, 157, 159, 163, 166, 169, 174, 179, 180, 190, 194, 195, 201, 232, 234, 240, 244, 247, 249, 251, 252, 257–260, 263, 264, 269, 289, 290, 293, 305, 306, 308, 312, 480, 486, 495, 497, 500, 507, 509–511, 515, 518, 519, 524, 529, 532, 543, 548, 550, 551, 559, 568, 576, 578, 582, 583, 586, 593, 596, 598, 631, 639, 648, 650, 652, 653], 24 case series [13, 320–323, 325–327, 332, 333, 335, 338, 348, 354, 356, 363, 371, 374, 375, 380, 383, 385, 390] and five case–control studies [407, 409, 416, 449, 450]. Among the most frequent study types were 48 documents that focused on surgical complications [13, 25, 45, 58, 69, 73, 77–80, 84, 85, 88, 101, 110, 125, 152, 157, 159, 163, 166, 174, 180, 194, 195, 234, 240, 252, 257, 264, 269, 289, 290, 306, 312, 327, 354, 356, 409, 416, 480, 500, 509, 529, 550, 568, 596, 639], 27 on implant survival [64, 67, 122, 154, 179, 232, 251, 260, 293, 308, 321, 371, 385, 495, 507, 510, 511, 515, 518, 519, 524, 532, 551, 582, 583, 631, 648], 17 on postoperative clinical outcomes [62, 95–97, 106, 112, 114, 120, 134, 190, 247, 305, 348, 375, 380, 497, 559] and 15 on PROMs and QALYs [81, 92, 99, 100, 127, 129, 135, 137, 244, 263, 449, 548, 576, 593, 636]. Besides, ten studies evaluated the patients’ mortality rate [31, 60, 76, 258, 259, 320, 323, 333, 652, 653], seven different implant types [118, 126, 201, 325, 332, 586, 598] and seven the long-term postoperative outcomes [82, 93, 94, 322, 383, 407, 543]. The “Trent” JAR published ten studies from 1997 to 2018 [9, 276], including nine cohort studies [9, 20–22, 27, 53, 218, 256, 276] and one case series [329]. Postoperative complications, short and long-term clinical outcomes, PROMs and QALYs, the efficacy of specific implants, comparison of different surgical techniques and survival analyses were among the main outcome of the published studies. The “Register of Orthopaedic Prosthetic Implant (RIPO) of Emilia-Romagna region” published 30 papers from 2002 to 2022 [24, 645]. Among them, twenty-eight were cohort studies [24, 26, 39, 43, 57, 145, 164, 189, 231, 288, 297, 482, 491, 502, 506, 508, 527, 540, 562, 563, 569, 585, 592, 597, 599, 624, 630, 645], one was RCT [476] and one registry protocol [468]. Of these 30 studies, 16 were survival [26, 164, 189, 231, 288, 482, 491, 502, 508, 527, 540, 563, 569, 585, 592, 599], six analysed implant types [24, 43, 297, 506, 624, 645], and two studied postoperative complications [597, 630]. Long-term postoperative outcomes [562], cost [57] and risk factors analysis [39], genetic studies [476], a protocol for registry study [468] and comparison of different surgical techniques [145] were among the primary outcomes of other study types.

**Table 4:** Hospital-based Arthroplasty Registry with more than one publication

JAR	Country	Lifetime (first—last year publication)	Number of papers	Number of papers/lifetime
THR RG HS for Special Surgery	USA	11 (2011–22)	89	8.09
Mayo Clinic TJR, Rochester	USA	25 (1997–22)	147	5.88
Partners Arthroplasty RG Massachusetts (PAR)	USA	1 (2020–21)	4	4.00
Singapore General HS Joint RG	Singapore	9 (2013–22)	33	3.67
Joint RG,Center for Advanced Orthopedics Larkin, Miami	USA	1 (2015–16)	3	3.00
Joint RG Uni Connecticut Health Center, Farmington	USA	1 (2018–19)	3	3.00
Endoprothesenregister in Regensburg Uni	Germany	2 (2016–18)	5	2.50
Michigan Arthroplasty RG Collaborative Quality Initiative	USA	6 (2016–22)	14	2.33
Schulthess local Shoulder Arthroplasty RG (SAR)	Switzerland	4 (2017–21)	8	2.00
American Hip Institute RG, Westmont, Illinois	USA	3 (2017–20)	6	2.00
North West Regional Arthroplasty Register Wrightington HS	UK	1 (2004–05)	2	2.00
New England Baptist HS RG, Tufts Medical Center, Massachusetts	USA	6 (2016–22)	11	1.83
Joint Implant Surgeons Practice RG, Ohio	USA	6 (2013–19)	10	1.67
Joint Replacement RG Uni of Manitoba	Canada	3 (2016–19)	5	1.67
St. Vincent’s HS Melbourne (SMART)	Australia	11 (2011–22)	18	1.64
Register of Orthopaedic Prosthetic Implant (RIPO) of Emilia-Romagna	Italy	20 (2002–22)	30	1.50
Total Joint RG Barnes-Jewish HS Washington Uni, Missouri	USA	8 (2014–22)	12	1.50
Joint RG Lausanne Uni HS—CHUV	Switzerland	2 (2020–22)	3	1.50
Total Joint Replacement RG Minnesota Uni, Minnesota	USA	2 (2013–15)	3	1.50
Shoulder Arthroplasty RG Fondren Orthopedic Research Institute (FORI), Texas	USA	6 (2015–21)	8	1.33
TJA RG Kentucky University, Lexington	USA	6 (2014–20)	8	1.33
Arthroplasty RG Thessaloniki (ART)	Greece	4 (2018–22)	5	1.25
RG Queen Mary HS	Hong Kong	5 (2016–21)	6	1.20
Geneva Arthroplasty RG (HUG)	Switzerland	12 (2010–22)	13	1.08
Local TKA RG Kantonsspital St. Gallen	Switzerland	7 (2014–21)	7	1.00
California Joint Replacement RG (CJRR), California	USA	6 (2014–20)	6	1.00
Rush Uni (Shoulder) Registry Chicago	USA	5 (2016–21)	5	1.00
HS Universitario Fundación Santa Fe de Bogotá	Colombia	4 (2017–21)	4	1.00
Replacement RG OrthoVirginia, Virginia	USA	2 (2018–20)	2	1.00
Institutional Joint RG in Hôpital Edouard Herriot	France	2 (2016–18)	2	1.00
TJA RG Texas Southwestern Uni, Texas	USA	2 (2016–18)	2	1.00
Department of Orthopedics Södersjukhuset	Sweden	2 (2013–15)	2	1.00
Toronto Western HS Joint Replacement RG	Canada	12 (2008–20)	11	0.92
Catalan Arthroplasty Register (RACat)	Spain	6 (2014–20)	5	0.84
Harris Joint RG Massachusetts General HS	USA	19 (2001–20)	15	0.79
Freeman Joint RG	UK	8 (2013–21)	6	0.75
Ontario Joint Replacement RG (OJRR)	Canada	4 (2006–10)	3	0.75
HealthEast Joint RG, Minnesota,	USA	10 (2003–13)	7	0.70
Arthroplasty RG Copenhagen Uni HS	Denmark	3 (2018–21)	2	0.67
Arthroplasty RG Uni California, San Francisco,	USA	3 (2017–20)	2	0.67
Italian Arthroplasty RG (RIAP)	Italy	9 (2012–21)	5	0.55
OrthoCarolina H&K Center, Charlotte,	USA	9 (2009–18)	5	0.55
SoFCOT Group (Socie ´te ´ Franc ¸aise de Chirurgie Orthope ´dique et Traumatologique)	France	14 (2008–22)	7	0.50
Chang Gung Memorial HS Joint RG, Taiwan	China	8 (2012–20)	4	0.50
Retrieved Orthopedic Implant RG Beaumont Health	USA	8 (2011–19)	4	0.50
Regional Joint RG Tauranga Public HS	New Zealand	6 (2013–19)	3	0.50
Institutional RG St Michael’s HS, Toronto Uni	Canada	4 (2016–20)	2	0.50
Total Joint RG Mount Sinai, NY	USA	4 (2015–19)	2	0.50
Register Kantonsspital Baselland Liesta	Switzerland	4 (2013–17)	2	0.50
Avon Knee RG	UK	4 (2003–07)	2	0.50
Trent Leicester	UK	21 (1997–2018)	10	0.48
Institutional RG SouthWest London Elective Orthopaedic Centre,	UK	16 (2006–22)	7	0.44
Institutional H&K arthroplasty RG St Olavs HS	Norway	7 (2015–22)	3	0.43
RG General HS KAT	Greece	7 (2015–22)	3	0.43
Arthroplasty Register Tirol Landeskrankheitstalten GmbH, Innsbruck	Austria	5 (2015–20)	2	0.40
Dresden Hip Surgery Registry	Germany	6 (2010–16)	2	0.33
Thomas Jefferson Uni HS, Arthroplasty RG Rothman Institute of Orthopaedics, Philadelphia	USA	16 (2006–22)	5	0.31
Implant RG Cleveland Clinic Foundation, Ohio	USA	14 (2003–17)	4	0.28
ORTHOTEP RG Uni HS Carl Gustav Carus	Germany	8 (2013–21)	2	0.25
Arthroplasty RG Balgrist Uni HS Zürich	Switzerland	10 (2012–22)	2	0.20

The JARs are listed based on the frequency of their publications to their lifetime

*H&K* Hip and Knee, *HS* Hospital, *RG* Registry, *S&E* Shoulder and Elbow, *THR* Total Hip Registry, *TJA* Total Joint Arthroplasty, *TJR* Total Joint Registry, *TKA* Total Knee Arthroplasty, *TKR* Total Knee Registry, *Uni* University

Concerning the publications’ frequency (number of publications/years of the JAR’s operation), the "THR Registry in Hospital for Special Surgery" is in the first place, with more than eight publications per year (89 papers from 2011 to 2022). "Mayo Clinic Total Joint Registry" is in second place with almost six publications per year (149 papers in twenty-five years), followed by the "Partners Arthroplasty Registry Massachusetts (PAR)" with four publications per year (4 articles in one year). More details are shown in Table 4.

### Countries with Level I and Level IV arthroplasty registries

Nine countries have national (Level I) and institutional (Level II–IV) JARs. In these countries, institutional JARs belong to the national JARs but publish their results independently. There are two national JARs in Oceania (Australian Orthopaedic Association National Joint Replacement Registry, New Zealand national joint registry) and six institutional JARs (Repatriation General Hospital, St. Vincent’s Hospital SMART, The Alfred Hospital, The Hollywood Hospital, Barwon in St John of God Hospital, Tauranga Public Hospital) The last one is distinct from but complementary to the New Zealand National Joint Registry. In Europe, six countries (Denmark, Germany, Norway, Sweden, Switzerland and the United Kingdom) own both Levels I and IV JARs. There is a national registry in Germany (Endoprosthesenregister Deutschland EPRD), and two out of four hospital-based registries (Registries at the University of Heidelberg and Regensburg University) work independently. In the United States of America, there are national JARs and 44 hospital-based or institutional registries. More details are shown in Tables 1, 2 and 3.

Some countries have only institutional JARs. Seven Asian and African countries (China, Hong Kong, Japan, Korea, Republic of China-Taiwan, Singapore and Tunisia) have hospital-based JARs. Still, no national JAR can be found on these continents (Table 1). Besides, seven European countries (Austria, France, Greece, Italy, Scotland, Spain, and Turkey) do not have national but only institutional JARs.

## Discussion

Our study mapped all Level II–IV JARs worldwide systematically based on their publications. Few hospital or institutional JARs have been found in Asia and Africa, with limited published studies. In Australia, Europe and the United States of America, all JARs levels can be found. The northern European countries (Scandinavia, United Kingdom) have well-known national JARs and institutional registries. In contrast, southern countries (Greece, Spain, Italy) lack a national but own hospital-based JARs publishing data. Due to financial, legal, and regulatory challenges in the United States of America [654], hospital-based JARs prevail, along with the existence of national registries: American Joint Replacement Registry (AJRR) and Kaiser Permanente. Most Level II–IV JARs are found in Europe and America. Some publish their data independently, while others also report through their relative national registries. All institutional or hospital-based JARs in the United Kingdom are part of the National Joint Registry. On the other hand, the "Endoprothesenregister in Regensburg University" and the "Endoprosthesis Register in Department of Orthopedic Surgery, University of Heidelberg" are institutional JARs that do not transfer data to the German national registry "Endoprothesenregister Deutschland (ERPD) ".

It is imperative to obtain a comprehensive inventory of institutional and hospital-based JARs, including Level II–IV, to thoroughly assess and categorize the patient-focused data they offer. The significance and necessity of these JARs can only be fully understood by identifying their quantity, location, and the variety and number of publications they generate. Therefore, we must prioritize acquiring this information to advance our understanding and improve patient care.

Hospital-based and institutional JARs worldwide reported all types of evidence-based pyramid studies. A few published studies are randomized clinical trials; most are cohorts, case-series and case–control studies. The published data are mainly retrospective, with the prospective studies being a minority. The study types differ among Level I and II–IV JARs. Level I national registries publish prospectively annual reports, including revision risk data for various implants. Level II–IV JARs collect more inclusive data to perform cohort and comparative studies; however, most are retrospective. Level I JARs data mainly control implant survival by monitoring the revision rate, the institutional performance and evaluating the quality offered pre-, intra- and postoperatively by all surgeons [8]. Level II–IV JARs data come mainly from senior high-volume surgeons and specialized centres worldwide and cannot be quickly adopted and generalized. However, Level II–IV JARs data are more inclusive. Demographics and baseline characteristics of patients, the type of implants, surgeons, surgical approaches and other procedural features and clinical and radiographical data are usually more detailed. Quality of patients’ life and medical complications other than implant failure as infections, deep vein thrombosis or pulmonary embolism, are also frequently recorded [63, 518, 655, 656]. The Harris Hip Score (HHS) and Hip disability and Osteoarthritis Outcome Score (HOOS) are used to report patients’ quality of life from most registries [2, 647]. There are also implant survival studies from various institutional JARs simultaneously reporting clinical outcomes, complications, PROMs and radiological implants’ data as secondary study outcomes [64, 321, 511, 524, 647]. Radiological data are beneficial to follow implants and understanding the reasons for failure, but they are only available by Level IV JARs [2, 116, 335, 450]. Several surgical approaches and comparative clinical studies of surgical outcomes between specialist orthopaedic surgeons and trainees have been evaluated [255, 428]. Level II–IV JARs often report studies that analyze risk factors (obesity/rheumatic diseases) of TJA outcomes, but also cost analysis studies of the length of hospital stay following TJA improving the cost-effectiveness of joint replacements [76, 158, 333, 425, 456, 486]. Genetic studies have also been performed [350, 476].

The lifetime and publishing frequency varies considerably between JARs. As previously mentioned, among the longest-running Level IV JARs are the "Mayo Clinic Total Joint Registry" from 1997 to 2022, followed by the "Trent" from 1997 to 2018 and the "Register of Orthopedic Prosthetic Implant (RIPO) of Emilia-Romagna Region" from 2002 to 2022. These JARs have published 147, 10 and 30 papers, respectively. The "Mayo Clinic Total Joint Registry" seems to be the oldest institutional registry and has published the most articles. However, the "Total Hip Registry in the Hospital for Special Surgery" is the JAR with the highest publishing frequency, with more than eight published papers per year, followed by the "Mayo Clinic Total Joint Registry" with almost six publications per year, and the "Partners Arthroplasty Registry Massachusetts (PAR)" with four publications per year. On the other hand, twenty-eight JARs publish less than one paper per year in their lifetime.

Besides, many institutional or hospital-based JARs have published only one article during their lifetime. This may be attributed to several factors. The patients and data enrollment of institutional registries is lower than the national ones, and a longer time is needed to complete and report studies. So, their lifetime may be longer than the actual measures from the first and last publication. However, the existence of some long-lasting low-frequency publishing JARs may be disputed in the future.

Our study has some limitations. The principal limit is that the institutional or hospital-based JARs data may not be fully accessible for several reasons. First, there are Level IV JARs, such as the “ German Orthopaedic Foot and Ankle Association's (D. A. F.)” registry [657], that only publish studies in their native language. Thus they are not included in this report. Secondly, only a few regional and hospital-based JARs manage a website to publish annual reports, such as national JARs, due to a lack of funding. Thirdly, most Level IV JARs do not have yearly reports available. If the reports are available online, they are not open to the public, contrary to national JARs [654]. Lastly, many Level IV JARs publish studies only once or twice in their lifetime [21, 24]. That way, a lot of helpful information may be lost.

## Conclusion

To our knowledge, this is the first systematic review mapping all institutional or hospital-based JARs worldwide. Most of these registries are found in Europe and America, reporting all types of evidence-based pyramid studies. The reported studies may have data missing from national registry reports as radiographic data, but they are often retrospective. The frequency of data reporting varies considerably among Level II–IV JARs, but this is generally not systematic. Their contribution is undeniable, mainly due to the detailed and variable data they collect. Further studies are needed to evaluate the quality of the offered knowledge in the clinical setting, especially for Level IV registries that do not publish their data annually or in a non-English language.
